# Physical Activity-Related Health Competence, Physical Activity, and Physical Fitness: Analysis of Control Competence for the Self-Directed Exercise of Adolescents

**DOI:** 10.3390/ijerph17010039

**Published:** 2019-12-19

**Authors:** Stephanie Haible, Carmen Volk, Yolanda Demetriou, Oliver Höner, Ansgar Thiel, Gorden Sudeck

**Affiliations:** 1 Institute of Sport Science, University of Tübingen, Wilhelmstr. 124, D-72074 Tübingen, Germany; 2 Department of Sport and Health Sciences, Technical University of Munich, Georg-Brauchle-Ring 60/62, D-80992 Munich, Germany

**Keywords:** control competence, adolescents, fitness, physical activity, health literacy, physical literacy

## Abstract

(1) Background: Individuals have to effectively manage their physical activity in order to optimize the associated physical and psychological health benefits. Control competence allows the individual to structure and pace physical activity in a health-enhancing way. The concept was developed within a model of physical activity-related health competence, and is related to the concepts of health literacy and physical literacy. Therefore, the study firstly aimed to validate a self-report scale to measure the physical and psychological facets of control competence in adolescents. Secondly, relationships between control competence and its basic elements, knowledge and motivation, as well as between control competence, sport activity, and fitness, were investigated. (2) Methods: In two cross-sectional studies, ninth grade adolescents (study A: *n* = 794, 51% female; study B: *n* = 860, 52% female) were tested using self-report scales (study A and B), a test for health-related fitness knowledge (study B), and cardiovascular and muscular fitness tests (study B). (3) Results: Confirmatory factor analyses confirmed the two-factor structure of the self-report scale for control competence in studies A and B. In addition, the results of structural equation modeling in study B showed a relationship between motivation (via control competence) and sport activity, and a relationship between control competence and fitness. (4) Conclusion: The questionnaire extends the ability to assess control competence in adolescents. Moreover the findings support the importance of control competence in order to achieve health benefits through physical activity.

## 1. Introduction

For adolescents, habitual physical activity (PA) is considered to be an important source of physical, psychological, cognitive, and social health benefits [1,2]. Adolescence is a crucial phase for the acquisition of PA behaviors, which will often be practiced until adulthood [3]. However, population-based surveys have consistently shown that participation in PA decreases during adolescence [1]. Most adolescents around the age of 15 years do not meet the World Health Organization (WHO) recommendations for health-related PA, which suggest a minimum of 60 min of moderate to vigorous PA daily [1].

Relating to this, the WHO’s Global Action Plan on Physical Activity (GAPPA) 2018–2030 states that “quality physical education and supportive school environments can provide physical and health literacy for long-lasting healthy, active lifestyles” [4]. Therefore, “health literacy” and “physical literacy” have increasingly become topics of interest in recent health science and exercise science research [5,6].

Although in the GAPPA 2018–2030, the two terms “health literacy” and “physical literacy” are combined as “health and physical literacy”, both terms have their own conceptual roots and diverse meanings. In recent years, intense efforts have been made to consensually elaborate on the concepts of both health literacy [7,8,9] and physical literacy [10]. Health literacy comprises knowledge, motivation, and competencies to access, understand, appraise, and apply information, in order to make judgements and decisions that positively affect health and well-being [7]. Within health literacy concepts, PA is rarely explicitly addressed. For example, in a review by Fleary and colleagues, solely two studies considered the association between health literacy and PA as one of several health-promoting behaviors. By contrast, associations between health literacy and substance use as well as health-information seeking behavior were investigated in the majority of the evaluated studies [9]. Physical literacy studies have focused on maintaining physically active throughout life and described physical literacy as “the motivation, confidence, physical competence, knowledge and understanding to value and take responsibility for engagement in physical activities for life” [11,12]. Explicit references to health have been made in some widespread physical literacy approaches [13], but a health-related focus is not a common core of physical literacy concepts [10,14]. Recently, the potential for increased consideration of physical literacy in the field of public health has been highlighted [15] and explicit conceptual links between physical literacy and health have been proposed [16].

In accordance with these basic ideas, a model of “PA-related health competence” (PAHCO) was developed to address the intersection of health literacy and physical literacy [17]. It integrates individual competencies to promote a healthy, physically active lifestyle, and combines health literacy and physical literacy concepts within a functional pragmatic understanding of competence. In this model, “control competence” is of particular importance, since it plays a central role in the self-directed structuring and pacing of PA in a health-enhancing way. In particular, it empowers a person to make judgements and decisions, not only to increase the quantity but also the quality of PA in terms of its beneficial effects for health and well-being [17,18]. Therefore, control competence establishes an eligible link between the concepts of health literacy and physical literacy by focusing on processing and applying health-related information in order to optimize health-enhancing PA behavior.

In adults, Sudeck and Pfeifer [17] have already developed and validated a questionnaire for sub-competencies of PAHCO. They also applied the questionnaire to investigate control competence and its impact on PA behavior and motor function in adults participating in exercise programs in primary prevention as well as rehabilitation settings. These findings supported the assumption that control competence was not only related to the quantity of PA, but also showed further associations with positive health outcomes. Hence, the assessment of control competence extends the possibility of empirically investigating the effectiveness of health-related exercise interventions. First, it covers domain-specific aspects of health literacy and therefore potentially allows us to better display effects of interventions. Second, factors which have not yet been covered in current physical literacy assessments can be addressed.

Therefore, the purpose of the present study was to examine whether the self-report questionnaire to assess control competence, previously used for adults [17], can be applied to adolescents, and whether control competence in adolescents is linked to PA behavior and health outcomes. In order to frame the outlined questions, the PAHCO model was used as the theoretical framework, which will be introduced before we specify our research questions.

### 1.1. Introduction to the PAHCO Model

The PAHCO model was based on the question of what demands individuals face in the context of achieving a healthy, physically active lifestyle [17]. In this respect, competence was regarded as a person’s ability to cope with challenges in particular situations, developed through learning processes and experiences gained from relevant context-specific demanding situations [19,20]. This functional pragmatic understanding of competence has found widespread applications in the assessment of educational outcomes and in educational research in general [19].

**Figure 1:**
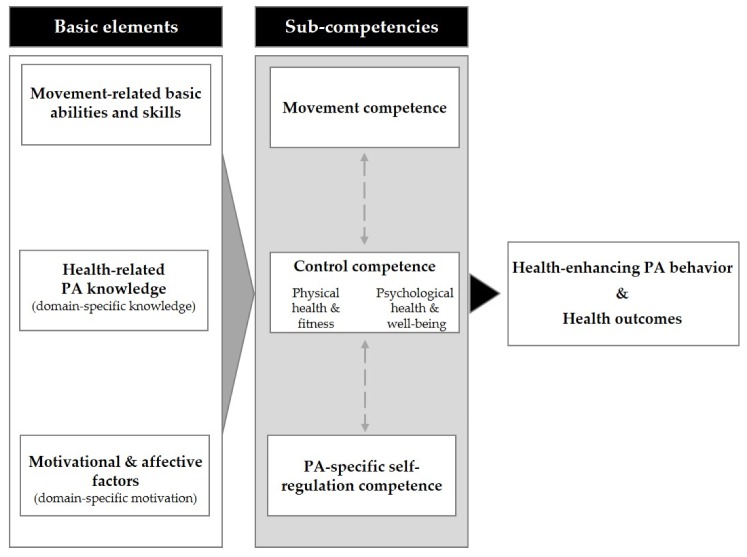
Model of physical activity (PA)-related health competence (PAHCO) [17].

Besides control competence, PAHCO includes the sub-competencies of “movement competence” and “self-regulation competence” (see Figure 1). One assumption of the PAHCO model is that each of these sub-competencies can specifically help in coping with the demands that arise during the initiation and maintenance of health-enhancing PA [17]. Individuals with high movement competence can adequately meet the motor demands of health-enhancing PA, including exercise and sport activities. People with high control competence can gear their own PA to optimize health benefits and minimize health risks. A person with high self-regulation competence can ensure the required regularity of health-enhancing PA. This sub-competence is most strongly related to motivational and volitional determinants of PA behavior, which are described in social-cognitive theories of health behavior and which are often empirically applied to PA behavior (e.g., the Theory of Planned Behavior [21]).

A fundamental idea of the PAHCO model is that the sub-competencies comprise basic motor, cognitive, and motivational elements. In line with the functional-pragmatic understanding of competence [19,22] as well as certain health literacy concepts [23], action-related competencies are characterized by the integration of basic elements such as domain-specific knowledge, skills, and motivation.

### 1.2. Distinguishing the Two Facets of Control Competence

It is assumed that different facets of control competence can be distinguished with regard to biopsychosocial health [17]. According to Franke [24], the objective and subjective dimensions of health are differentiated. One facet can be assigned to an objective biomedical health concept relating to physical health and fitness. This means that individuals are empowered to regulate and manage their exercise in a health-competent way, and are therefore able to independently estimate their exercise intensity and self-direct their PA in order to achieve an adequate stimulus to promote their own physical health [18]. The second facet relates to the subjective aspect of health, which places greater consideration on the subjective-affective experience of exercise [25,26]. This means that although exercise might be paced and regulated adequately to achieve effective physical health benefits, the affective response to exercise might not be positive in the same way; therefore, according to a biopsychosocial health approach, it is also important that individuals are able to regulate exercise according to its psychological health benefits and subjective well-being [27].

Previous empirical investigations with adults have underlined these assumptions: The two facets of control competence for physical training (the biomedical health concept) as well as for PA-specific affect regulation (the subjective health concept) were differentiated in confirmatory factor analyses [17]. For adolescents, however, no empirical studies have tested the assumption that facets of control competence should be differentiated with regard to their biomedical and subjective health dimensions.

### 1.3. From Basic Elements, via Control Competence, to Health Outcomes

Focusing on one facet, control competence for physical training is based on skills in perceiving exertion, pacing physical training, and applying training and knowledge in a health-enhancing way [17]. For instance, compared to physical literacy approaches, this means that individuals have knowledge and understanding of health-related physical fitness and appropriately apply this knowledge to physical training situations [10,28]. Individuals can also use their body signals to regulate the degree of physical strain, be aware of their physical state during PA, and use these to pace their exercise and understand how physical training can improve health-related endurance and strength [18]. To achieve this, motivational and affective factors, such as confidence in the ability to structure and control exercise comparable to task self-efficacy [29] or perceived behavioral control [21], are beneficial [17]. Furthermore, as in physical literacy approaches, positive attitudes and interest regarding PA and health are considered to be conducive to the development of control competence for physical training [10,30]. The joint application of these elements enables individuals to pace their PA appropriately, avoiding excessive, insufficient, or incorrect load in variable PA situations. An initial study showed [17] that control competence for physical training was associated independently with physical fitness, even if the impact of PA behavior on physical fitness was controlled; therefore, control competence was directly associated with PA and exercise, and increased the level of the respective behavior. Additionally, control competence was shown to be positively related to the quality of PA and its effects in terms of optimizing physical health benefits [17].

There is a lack of empirical evidence that elaborates on the relationships between the basic elements—control competence, PA behavior, and health benefits—in adolescents. This deficiency can be found in the research areas of both health literacy and physical literacy, where the relationships between underlying knowledge, skills, and abilities, and particular associations with health behavior and health outcomes, have rarely been explored in adolescents [6,31,32].

### 1.4. Aims and Hypotheses

Based on the outlined theoretical considerations, two research questions were proposed for the present study. First, we wanted to establish for adolescents whether the self-report scale for control competence is an appropriate measure for distinguishing between the two facets of control competence for physical training and PA-specific affect regulation. Second, we focused on the facet of control competence for physical training. Thus, we wanted to analyze the outlined theoretical associations between domain-specific knowledge, domain-specific motivation, and control competence for physical training, sport activity, and health-related physical fitness. Henceforward, we use the term “sport activity” according to the definition used within the assessment approach for PA, exercise, and sport activity of Fuchs and colleagues [33]. It takes into account German-speaking particularities in the delimitation of different forms of PA. In applying the term “sport activity”, we included exercise and sport activities in a broader sense done out of for example, social, personal or health-related reasons (e.g., running, strength training, dancing, recreational swimming) as well as sports in a narrower sense with predominant characteristics of competition and performance orientation (for example, soccer, track and field activities, basketball, swimming), which are typically organized in sports clubs or partly self-organized in leisure activities.

The following hypotheses were tested:

(1) Domain-specific knowledge and domain-specific motivation are positively associated with control competence for physical training.
(2) Control competence for physical training mediates the association between domain-specific knowledge and domain-specific motivation and sport activity.
(3) Control competence for physical training is related to health-related physical fitness, controlled for the level of sport activity.

## 2. Methods

To answer the research questions, data from two cross-sectional studies (A and B) of adolescents were used. For research question 1, we used both samples to replicate the results. Research question 2 was answered using the sample for study B.

### 2.1. Participants and Procedure

#### 2.1.1. Study A

In a cross-sectional study, 794 ninth grade students (girls: 402 (50.6%); boys: 392 (49.4%)), with a mean age of 14.3 years (Standard Deviation (SD) = 0.5), completed a paper-and-pencil test in the fall of 2015. We recruited the participating classes from secondary schools in the Tübingen district (approximately 1.8 million inhabitants) in the German federal state of Baden-Württemberg. To reach a minimum level of students for validation of several measures, we drew a sample of 42 out of 98 secondary schools. We attempted to evenly spread these schools over four areas of the Tübingen district and different school types. School boards sent information about the study and data protection to school principals and responsible PE teachers. Teachers from 22 schools responded (participation rate: 52.3%), signaling their interest in the study. Generally, two to three classes (due to class organization) per school took part. Written informed consent to participate in the study was obtained from all adolescents and their parents. Approval was obtained from the Ethics Committee at the Faculty of Economics and Social Sciences, University of Tübingen (A2.5.4-059_aa), and the Regional Council of Tübingen.

#### 2.1.2. Study B

The data of study B employed baseline measures drawn from the GEKOS cluster randomized controlled trial [34]. Briefly, 860 ninth grade students (girls: 449 (52.2%); boys: 411 (47.8%)), with a mean age of 14.2 years (SD = 0.5), took part in this study. We recruited classes through the school boards in Baden-Württemberg, who informed school principals and responsible PE teachers about the study. From September 2017 to April 2019, the students were tested with a paper-and-pencil test and a physical fitness test during regular school lessons. We obtained approval for the study from the Ethics Committee for Psychological Research at the University of Tübingen (Revision_1_ 2017_0825_78), the Regional Council of Tübingen, and the Ministry of Education and Cultural Affairs in the federal state of Baden-Württemberg. Written informed consent was given by the students and their parents to complete the tests.

### 2.2. Data Collection

In study A and study B students completed written tests during regular school classes (90 min). In study B an additional physical fitness test was carried out in physical education classes (90 min). In both studies trained researchers collected and entered data. Standardized test manuals were used and all procedures as well as deviations from test manual during data collection were documented in documentation forms.

### 2.3. Measures

Descriptive statistics for control competence items of study A and B are shown in Supplementary Materials, Table S1. Mean values, standard deviation, and bivariate correlations of study B variables are shown in Supplementary Materials, Table S2.

#### 2.3.1. Facets of Control Competence (Studies A and B)

Facet 1, control competence for physical training (CCPT) was measured by six Likert-Scale items. To assess facet 2 (PA-specific affect regulation (PAAR)), four Likert scale items were used. The Likert scale ranged from totally disagree (1) to totally agree (5) and was modified from the original four to five answer options to ensure consistency across the questionnaires [17]. The items addressed the application of training-specific knowledge of actions, and the usage of the perception of body signals and perceived exertion to pace and structure exercise and training, targeting either endurance and strength (CCPT; e.g., *“I can use my body signals (pulse, breathing speed) very well to gauge and regulate the amount of physical load”*; Cronbach’s α_A_ = 0.77; Cronbach’s α_B_ = 0.78) or mood, distraction, and stress regulation (PAAR; e.g., *“I am well able to work off pent-up stress and inner tension through exercise”*; Cronbach’s α_A_ = 0.85; Cronbach’s α_B_ = 0.88). All English-translated and German anchors for control competence items, as well as the descriptive statistics for studies A and B, are shown in Supplementary Materials, Table S1.

#### 2.3.2. Domain-Specific Knowledge (Study B)

Domain-specific knowledge was assessed using a health-related fitness knowledge test, which we developed in the context of study B [34]. The performance test contained 27 complex multiple choice, matching, and sorting items and open-ended questions [35]. The test addressed knowledge of the principles of exercise and physical fitness, knowledge about risk reduction and the prevention of injuries related to PA and exercise, and knowledge about the health benefits of PA. The person parameters were obtained using weighted likelihood estimation (WLE) [36]. The WLE person separation reliability of the test was 0.65.

#### 2.3.3. Domain-Specific Motivation (Study B)

To assess domain-specific motivation, scales to measure health and fitness-related attitudes and interest were applied. Attitudes towards the health effect of PA, which were already used in previous studies with adolescents, were assessed with four affective items (e.g., *“I feel better and healthy after being physically active”*; Cronbach’s α = 0.72) and three cognitive items (e.g., *“regular exercise is healthy”*; Cronbach’s α = 0.61) [37]. Interest in training, physical fitness, and health was measured by four items (e.g., *“I’m interested in learning about fitness and health”* or *“I generally have fun to engage myself with how to do endurance, muscle, and flexibility training”*; Cronbach’s α = 0.79), which were developed based upon the Programme for International Student Assessment (PISA) 2006 [34,38]. For this analysis, we built a latent factor for health- and fitness-related motivation, based on the three scale mean values for cognitive attitude, affective attitude, and health- and fitness-related interest.

#### 2.3.4. Sport Activity (Study B)

The Physical Activity, Exercise, and Sport Questionnaire (BSA-F: derived from German: Bewegungs- und Sportaktivität Fragebogen) was used to measure habitual exercise and sport activities [33]. The students could indicate up to four exercise and sport activities that they normally undertook per week, as well as the frequency per week and the duration in minutes of each episode. Based on our own previous pilot study, the answer relating to frequency per month was adjusted to frequency per week. Out of four possible activities, including their frequency and duration, an index was built for habitual weekly level (in hours) of habitual exercise and sport activities.

#### 2.3.5. Health-Related Physical Fitness (Study B)

Health-related physical fitness was assessed by measuring indicators of cardiorespiratory (endurance) and muscular (strength endurance) fitness, which are associated with several health outcomes in adolescents [39]. A 20-m shuttle run was used to assess cardiorespiratory fitness [40,41]. For this, the protocol of secondary schools in the federal state of Baden-Württemberg, Germany was used. It starts at 8.0 km/h, increasing on 9 km/h after a minute and afterwards by 0.5 km/h every minute, comparable to the Eurofit protocol [42]. Results are reported at the running speed (km/h) at the last completed stage and z-scores were calculated using age- and sex-specific values [43]. To assess strength endurance, three strengthening exercises (standing long jump, push-ups (40 s), and sit-ups (40 s)) of a standardized physical fitness test were used (Deutscher Motorik-Test 6-18 (DMT 6-18) [44]. Number and length were also z-standardized by age and sex, using national reference data, and combined to a muscular fitness score. Based on these measures, a latent factor for health-related physical fitness, with indicators of cardiorespiratory and muscular fitness, was built.

### 2.4. Statistical Analyses

Statistical analyses were conducted using SPSS Version 25 (IBM, New York, NY, USA) and Mplus Version 8.3 [45]. In advance of the main analyses, descriptive and correlational analyses were carried out.

The main analyses were conducted using structural equation modeling with student level variables and school level (study A) and class level (study B) variables, respectively, considering the special features of nested data (in Mplus: type = complex). In order to replicate the two-factor structure of control competence in an adolescent sample, confirmatory factor analyses were applied in study A and study B, using a maximum-likelihood estimation. For convergent validity, factor reliability was calculated [46]. For discriminant validity, the Fornell–Larcker criterion was assessed [47]. Indicator reliability was analyzed using squared multiple correlation [46].

In order to test the outlined hypotheses regarding the relationships between domain-specific knowledge and motivation, control competence for physical training, sport activity, and health-related physical fitness, structural equation modeling was used to analyze the global model fit and local model parameters. The significance level for the local model parameters was set at 0.05. To assess the global model fit, we used the confirmatory fit index (CFI: acceptable ≥ 0.95; good ≥ 0.97), the root mean square error of approximation (RMSEA: acceptable ≤ 0.08; good ≤ 0.05), and the standardized root mean square residual (SRMR: acceptable ≤ 0.10; good ≤ 0.05) [48].

There was an average of 0.4% and 3.8% missing data with respect to the control competence items of studies A and B, respectively. For study B, 4.8% missing data occurred within the listed measures. The higher missing data rate in study B was caused by the fact that written and motor tests were conducted separately; 25 students (2.9%) missed the written test, and 58 students (6.7%) missed the motor test by not attending school or having an injury on the test days. Missing values for single items ranged from 0.1% (*n* = 1, cognitive attitude scale) to 1.2% (*n* = 12, e.g., cardiorespiratory fitness). The missing values were model-based replaced within the full information maximum likelihood (FIML) procedure of Mplus.

## 3. Results

### 3.1. Confirmatory Factor Analysis of Control Competence

The descriptive statistics, including skewness and kurtosis, as well as reliability indices for all the control competence items of studies A and B, are reported in Supplementary Materials, Table S1. In both studies the comparison of the one-factor to the two-factor model of control competence showed a significantly better fit (see Table 1) in favor of the two-factor model.

**Figure 2:**
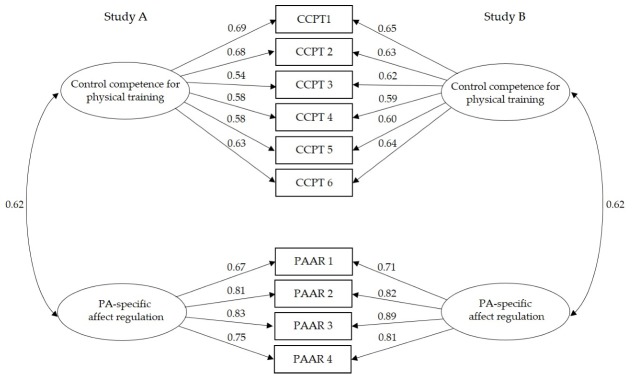
Results for the confirmatory factor analysis of the two-factor model of control competence (standardized path coefficients, all *p* < 0.01). PAAR: PA-specific affect regulation; CCPT: control competence for physical training.

The results for the local model parameters for the two-factor model are shown in Figure 2. The standardized factor loadings (indicator reliability) were statistically significant for all indicators of the two factors (*p* < 0.001) and ranged from 0.54 to 0.69 (study A) and 0.59 to 0.65 (study B) for CCPT, and 0.67 to 0.83 (study A) and 0.71 to 0.89 (study B) for PAAR; however, mixed results were obtained in terms of squared multiple correlations. In both studies, the indicators CCPT3 to CCPT5 fell below the cutoff value of 0.40. In terms of factor reliability (composite reliability, CR) for convergent validity, the two factors displayed good to very good values (CR_CCPT A/B_ = 0.79/0.79; CR_PAAR A/B_ = 0.85/0.88). The correlation between the two factors existed in both studies *r*_A/B_ = 0.62. In terms of discriminant validity (Fornell–Larcker Criterion), the average variance extracted (AVE) was higher for PAAR and equal for CCPT than the squared factor correlation between CCPT and PAAR (AVE_PAAR A/B_ = 0.59/0.66; AVE_CCPT A/B_ = 0.38/0.39). In summary, the global and local model fit of the CFAs were very similar in studies A and B.

### 3.2. Path Model for Control Competence for Physical Training

**Figure 3:**
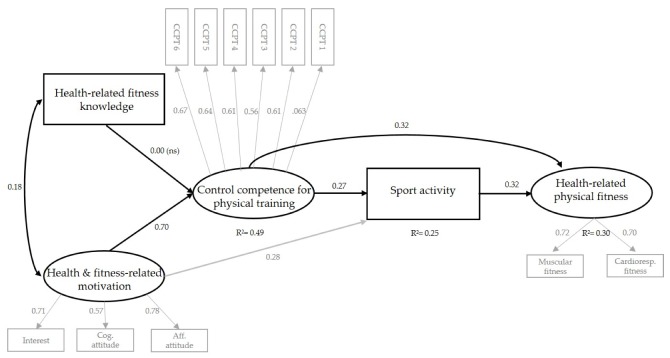
Path diagram of the model. Coefficients and factor loadings reported are standardized.

In study B, the variables were normally distributed, with skewness values of −1.35 to 1.18 and kurtosis values of −0.27 to 2.00 [49]. Descriptive results and bivariate correlations between all the variables in the model are shown in Supplementary Materials, Table S2. To address the second main hypothesis, a structural equation model was tested, based on the assumptions of control competence for physical training (see Figure 3). The global fit indices, except for CFI, had acceptable to good values: χ^2^_(60)_ = 209.348, χ^2^/df = 3.49, CFI = 0.94, RMSEA = 0.05, RMSEA 90% CI = 0.05–0.06, SRMR = 0.04. While there was a positive correlation between health- and fitness-related motivation and health-related fitness knowledge (β = 0.18, *p* < 0.001), only health- and fitness-related motivation was positively associated with control competence for physical training (β = 0.70, *p* < 0.001) in the path model. The bivariate correlation showed a small association between control competence for physical training and domain-specific knowledge (*r* = 0.10; *p* < 0.001; see Supplementary Materials, Table S2). The indirect effect of health- and fitness-related motivation, via control competence, for physical training on sport activity was significant (β = 0.19, *p* < 0.001), but there was no mediation between health-related fitness knowledge and sport activity via control competence for physical training (β = 0.00, *p* = 0.98). As hypothesized, control competence for physical training was positively associated with health-related physical fitness (β = 0.32, *p* < 0.001). The model explained 30% of the variance of health-related physical fitness and 49% of the variance of control competence for physical fitness.

## 4. Discussion

With respect to the increasing physical inactivity in economically developed countries [1,4], promoting individual competence to achieve a physically active lifestyle is still a significant challenge for health and exercise professionals. The present study provides empirical information on whether assumptions about control competence relating to health-enhancing PA are applicable to adolescents. The applied competence-oriented approach helps to overcome the predominance of quantitative outcome parameters in PA research that, for instance, address only the level of PA behavior. Instead, it focuses on the qualitative aspects of sport activity that is performed to optimize physical and psychological health benefits [17]; hence, control competence was the focus of the present study.

### 4.1. Distinguishing the Two Facets of Control Competence

The results for the two adolescent samples showed the favorability of a two-factor model for distinguishing control competence for physical training and PA-specific affect regulation. The similar results for both samples underlined the replicable nature of the studies. In addition to a good global model fit for the two-factor model, both factors could be satisfactorily delimited from each other. Medium correlations between both factors were a little higher than those of a study with adults (study A: *r* = 0.59; study B: *r* = 0.48 [17]).

With respect to the measurement models, the results indicated that the self-report scale would be suitable for adolescents aged 14 to 16 years. The indicators and factor reliability were good, and comparable to results for adults [17]; nevertheless, minor limitations must be made according to the discriminant validity of control competence for physical training. In particular, indicator reliabilities were low, as was also observed in validation studies with adults, especially with regard to the items targeting content other than aerobic activity. This was accompanied by a marginal Fornell–Larcker criterion for discriminant validity. Certainly, comparable to the study with adults [17], the findings prompt further consideration to balance the broadness of the construct, the number of items, and the delimitation of the two facets of control competence; nevertheless, the present version of the short questionnaire has provided a useful measure of the meaningfully distinct facets of control competence relating to physical health and fitness, as well as to subjective well-being, in adolescents. These results emphasized that the individual empowerment of adolescents, in relation to the structuring and pacing of exercise, should be addressed with regard to physical fitness and/or subjective well-being.

### 4.2. Associations between Basic Elements, Control Competence for Physical Training, Sport Activity, and Health-Related Physical Fitness

#### 4.2.1. Basic Elements, Control Competence for Physical Training, and Sport Activity

The assumption that control competence for physical training is related to domain-specific knowledge and motivation could be partially confirmed; however, the empirical findings of the path model only showed associations to health- and fitness-related motivation. Strong empirical associations indicated that interest in training, physical fitness, health, and positive attitudes towards the health effects of PA are associated with higher scores for control competence for physical training. The association between health- and fitness-related motivation and sport activity was not only mediated by control competence for physical training, but health- and fitness-related motivation was also directly associated with the level of behavior. In summary, our findings pointed toward the assumption that health- and fitness-related motivation might strengthen control competence for physical training and might increase sport activity. These findings were comparable with results based on the information–motivation–behavior skill (IMB) model [50]; for instance, Kelly and colleagues [51] found a positive relationship between personal motivation and behavioral skills, which in turn were related to PA in adolescents. Their findings supported the assumptions of the IMB model, which indicated that a well-motivated and well-informed person has essential objective und subjective behavioral skills to promote the initiation and maintenance of health-promoting behavior [50].

In contrast to our assumptions, domain-specific knowledge was not consistently associated with control competence for physical training. Although the correlation analyses showed at least a small association, in our path model, health-related fitness knowledge did not explain the variation in control competence for physical training beyond domain-specific motivation. However, we found a positive relationship between domain-specific motivation and knowledge, which was also found by Kelly and colleagues [51] in the IMB model. This small-to-medium association implied that higher health-related fitness knowledge supports positive attitudes towards health and fitness, and positive domain-specific attitudes and interest encourage the acquisition of health-related fitness knowledge.

To further interpret these findings, the conceptual and methodological aspects should be discussed. Our results did not confirm the competence-oriented assumption of a high-road integration of domain-specific knowledge and control competence. High-road integration means that knowledge and skills are connected by reflection in and/or on action and occurs in tasks that require thinking. Learners have to reflect on how to carry out a task and on available knowledge and skills to cope such a task [22,52]. However, the association between domain-specific motivation and domain-specific knowledge and domain-specific motivation and control competence for physical training indicated that, nonetheless, a high-road integration process may exist. In particular, high-road integration of attitudes includes that understanding why a certain attitude is useful in a specific context and the attitude of being willing and able to act critically is very important [22]; therefore, it could be that intense engagement and interest in a topic, such as health- and fitness-related attitudes and interest, might support understanding in the sense of high-road integration.

In our study, we did not find a positive association of knowledge and control competence for physical training with sport activity. Methodologically, knowledge testing has generally been criticized for rarely addressing the particular behavior of interest [53,54]. Even though in the development of the health-related fitness knowledge test we tried to address this problem, it was still difficult to determine an independent association between health-related knowledge and control competence and, thus, more competent behavior.

#### 4.2.2. Control Competence, Sport Activity, and Health-Related Physical Fitness

It was assumed that control competence for physical training is beyond the association between sport activity and health-related physical fitness, directly associated with health-related physical fitness. Our results showed a positive relationship between control competence for physical training and objective measured physical fitness. This association existed parallel to a direct relationship of control competence with sport activity, which in turn was positively correlated with health-related physical fitness. These findings confirmed the results of the validation study with adults, showing even higher path coefficients than in the adult study [17].

Further, in physical literacy concepts PA is not only discussed as an outcome of physical literacy, but also as a determinant to enhancing physical literacy [10,55]. In IMB research, Fisher and colleagues [50] went even further and stated that health outcomes might influence individuals’ future information, motivation, and behavioral skills according to a reciprocal relationship. In line with this, the correlational patterns in our study (see Supplementary Materials, Table S2) suggested further investigation of this reciprocal relationship, since the bivariate correlation analysis of our sample showed no significant correlation of health-related fitness knowledge with sport activity, but showed a correlation with health-related physical fitness. For these reciprocal relationships, our cross-sectional study can provide initial information, but cannot infer causality; therefore, it would be valuable to investigate the (reciprocal) relationships in longitudinal studies, further examining how PA and health outcomes might promote control competence and its basic elements.

### 4.3. Strengths and Limitations

This is the first investigation of adolescents regarding the facets of control competence and the associations of control competence with the basic elements, sport activity and physical fitness. One strength of our study was that we used two samples of adolescents with sufficient statistical power, so that we could replicate the results of the validation study for adults. To assess associations with control competence, we used a path model to obtain insight into important relationships between domain-specific knowledge and domain-specific motivation, and PA and health outcomes, in adolescents. Our findings regarding the direct association of control competence with health outcome indicators can add value to the concepts of physical literacy and health literacy, providing justification for further investigation of the underlying mechanism of control competence within the PAHCO model.

We used objective measures, such as the validated health-related fitness knowledge test and health-related physical fitness tests, for research question two in study B, whereas in the previous study with adults, motor function was assessed using standardized questionnaires [17]. Hence, we tried to prevent possible common method bias. Nevertheless, control competence for physical training and domain-specific motivation were assessed using self-report Likert scales, which may be the reason why the discriminability of the two constructs was rather low. Although self-report assessments are often used in health literacy research, the extent to which scores of the control competence scale go align with competent behavior is still open [17]. Therefore, future investigations might focus on developing measurements to objectively capture control competence.

Regarding the results relating to associations with control competence, a limitation is that physical fitness is only one indicator of physical health; therefore, it would be interesting to further evaluate health indicators for adolescents, which are also currently lacking in physical literacy research [16]. Furthermore, in study B our measurements regarding domain-specific knowledge, motivation, and health outcome predominantly addressed the biomedical health concept and consequently the facet of control competence for physical training. Therefore, with regard to the second facet, PA-specific affect regulation, indicators of psychological health and well-being as well as assessments for domain-specific knowledge and domain-specific motivation must be considered in future investigations. In adults, an ambulatory assessment study has already shown the moderating role of PA-specific affect regulation for the association between PA and affective well-being in everyday life [27].

In addition to domain-specific motivation and knowledge, physical capabilities and body awareness are also important basic elements of control competence; therefore, a more differentiated and complete analysis of these PA-specific elements should also be considered. For example, this could be achieved by measuring body interoception and awareness, for which objective instruments, such as interoceptive tasks, exist [56].

With respect to our sample, the generalization of the results is limited. Our sample included adolescents around the age of 15 years who were attending high school. It did not represent younger adolescents or adolescents with lower levels of education. Although for adolescents older than 17 years the questionnaire has already been applied in university sport [17], further investigations may need to address adolescents of different ages and educational and socio-economic status.

## 5. Conclusions

The purpose of the study was to examine the control competence of adolescents as a prerequisite for being regularly physically active in a health-enhancing way. Our findings showed that especially in times with insufficient PA among adolescents [1,4] it may be important to promote control competence, for example in physical education. Hereby, control competence can be considered as possible characteristic to establish a link between health literacy and physical literacy as it is addressed by the GAPPA. In adolescence—a crucial phase of health behavior development—pacing PA appropriately and avoiding excessive or insufficient load in PA situations can support the initiation and maintenance of self-directed health-enhancing PA [17,18]. The short self-report questionnaire allowed us to economically assess individual differences in adolescents regarding biomedical and affective facets of control competence. In addition, our results provided conceptual and empirical findings regarding the development of interventions to promote competencies for a physically active lifestyle, in order to empower individuals to positively align their PA behavior with biopsychosocial health. These investigations of adolescents, using the competence-based PAHCO approach posited by Sudeck and Pfeifer, supported the integration of control competence in exercise and health-related research, since this element has not been adequately represented in theories of physical literacy and in theories of health literacy [17].

Future studies are necessary to provide further information about the process of acquisition of domain-specific knowledge and domain-specific motivation and their role in the development of control competence [34]. Prospectively, in accordance with the GAPPA [4] and pedagogical considerations regarding the educative potential of PE [57] this might contribute to well-founded school-based interventions addressing the autonomous and self-directed exercise of adolescents, with the aim of promoting health-enhancing PA.

## Figures and Tables

**Table 1:** Goodness of fit statistics for the one- and two-factor models of control competence.

Models	χ^2^	*p*(*df*)	χ^2^/df	CFI	RMSEA	90% CI	SRMR
Study A: 1 factor	421.58	<0.001 (35)	12.05	0.82	0.12	0.11, 0.13	0.08
Study A: 2 factors	67.78	<0.001 (34)	1.99	0.98	0.04	0.02, 0.05	0.03
Study B: 1 factor	510.50	<0.001 (35)	14.59	0.83	0.13	0.12, 0.14	0.09
Study B: 2 factors	74.09	<0.001 (34)	2.18	0.99	0.04	0.03, 0.05	0.03

**Note**. df = degrees of freedom; CFI = comparative fit index; RMSEA = root mean square error of approximation; CI = confidence interval; SRMR = standardized root mean square residual.

## Supplementary Materials

The following are available online at https://www.mdpi.com/1660-4601/17/1/39/s1, Table S1: Descriptive statistics for the individual items of control competence for studies A and B (English-translated and German versions). Table S2: Mean values, standard deviation, and bivariate correlations of the relevant variables. File S3: Data study A. File S4: Data study B. File S5: Explanation variable data study A and B.
