# Supplementary material for: Physical Activity-Related Health Competence, Physical Activity, and Physical Fitness: Analysis of Control Competence for the Self-Directed Exercise of Adolescents
Source: Int J Environ Res Public Health. 2019 Dec 19;17(1):39. doi: 10.3390/ijerph17010039 (PMC6981936; doi:10.3390/ijerph17010039)
Supplement: Supplementary file 1 [file ijerph-17-00039-s001.zip › Supplementary Material_PAHCO/Supplementary Material_File S5.pdf]

**Supplementary Material**

**File S5.** Explanation variables study A and study B

| Study A | | |
|---|---|---|
| **Variable** | **Explanation** | **Values** |
| Laufnr | consecutive number of test person | |
| gender | gender | 1 = female; 2 = male |
| school | cluster variable | 1-22 |
| Alter | age | |
| SKT1 | Item control competence for physical training 1 | 1-5 |
| SKT2 | Item control competence for physical training 2 | 1-5 |
| SKT3 | Item control competence for physical training 3 | 1-5 |
| SKT4 | Item control competence for physical training 4 | 1-5 |
| SKT5 | Item control competence for physical training 5 | 1-5 |
| SKT6 | Item control competence for physical training 6 | 1-5 |
| BR1 | Item physical activity-specific affect regulation 1 | 1-5 |
| BR2 | Item physical activity-specific affect regulation 2 | 1-5 |
| BR3 | Item physical activity-specific affect regulation 3 | 1-5 |
| BR4 | Item physical activity-specific affect regulation 4 | 1-5 |
| SKT | Mean score control competence for physical training | |
| BR | Mean score physical activity-specific affect regulation | |
| | | |

| Study B | | |
|---|---|---|
| **Variable** | **Explanation** | **Values** |
| Laufnr | consecutive number of test person | |
| sex | gender | 0 = female; 1 = male |
| class | cluster variable | 1-48 |
| Alter | age | |
| T1CCPL1 | Item control competence for physical training 1 | 1-5 |
| T1CCPL2 | Item control competence for physical training 2 | 1-5 |
| T1CCPL3 | Item control competence for physical training 3 | 1-5 |
| T1CCPL4 | Item control competence for physical training 4 | 1-5 |
| T1CCPL5 | Item control competence for physical training 5 | 1-5 |
| T1CCPL6 | Item control competence for physical training 6 | 1-5 |
| T1PAMR1 | Item physical activity-specific affect regulation 1 | 1-5 |
| T1PAMR2 | Item physical activity-specific affect regulation 2 | 1-5 |
| T1PAMR3 | Item physical activity-specific affect regulation 3 | 1-5 |
| T1PAMR4 | Item physical activity-specific affect regulation 4 | 1-5 |
| T1CCPL | Mean score control competence for physical training | |
| T1PAMR | Mean score physical activity-specific affect regulation | |
| | | |
| T1HrFK | WLE Score health-related fitness knowledge | |
| T1FG | Mean score interest in training, physical fitness, and health | |
| T1ATTK | Mean score cognitive attitudes towards the health effects of PA | |
| T1ATTA | Mean score affective attitudes towards the health effects of PA | |
| T1pA | weekly duration (in hours) of habitual physical activity | |
| T1zSR | Shuttle Run: z-standardised (age and gender [43]) values of running speed (km/h) at the last completed stage | |
| T1zStr | mean of three z-standardised strength exercises (age and gender [44]) | |
