# Supplementary material for: Physical Activity-Related Health Competence, Physical Activity, and Physical Fitness: Analysis of Control Competence for the Self-Directed Exercise of Adolescents
Source: Int J Environ Res Public Health. 2019 Dec 19;17(1):39. doi: 10.3390/ijerph17010039 (PMC6981936; doi:10.3390/ijerph17010039)
Supplement: Supplementary file 1 [file ijerph-17-00039-s001.zip › Supplementary Material_PAHCO/Supplementary Material_Table S1.pdf]

Supplementary Material

Table S1. Descriptive statistics for the individual items of control competence for studies A and B (English-translated and German versions).

Abbr.	Item		*M*	*SD*	*S*	*K*	r_{it}	α *(A/B)*
CCPT1	I am able to adjust my training effort well to my physical condition.	A	3.80	0.91	-0.55	0.11	0.55	
	Ich bin in der Lage eine Trainingsbelastung gut auf meine körperlichen Voraussetzungen anzupassen.	B	3.77	0.86	-0.47	0.03	0.54	
CCPT2	I know how to use physical training to improve my endurance in the best possible way.	A	3.77	0.94	-0.60	0.07	0.56	
	Ich weiß, wie ich mit körperlichem Training meine Ausdauerleistung am besten steigern kann.	B	3.64	0.98	-0.46	-0.30	0.55	
CCPT3	If my muscles are tensed up, I know exactly how to counter this through physical activity.	A	2.90	1.20	0.09	-0.91	0.47	
	Wenn ich muskulär verspannt bin, weiß ich genau, wie ich mit körperlicher Aktivität etwas dagegen tun kann.	B	2.81	1.18	0.22	-0.82	0.54	
CCPT4	I can use my body signals (pulse, breathing speed) very well to gauge and regulate the amount of physical load.	A	3.23	1.16	-0.18	-0.84	0.53	0.77/ 0.78
	Ich kann Signale meines Körpers (Puls, Atemgeschwindigkeit) sehr gut dafür nutzen, um die Höhe der körperlichen Belastung einzuschätzen und zu regulieren.	B	3.21	1.12	-0.22	-0.78	0.50	
CCPT5	If I want to enhance my health by strengthening trunk muscles (back, stomach), I am confident that I know the right exercises to do.	A	3.61	1.12	-0.50	-0.53	0.52	
	Wenn ich meine Gesundheit durch die Kräftigung der Rumpfmuskulatur (Rücken, Bauch) fördern möchte, traue ich mir zu, die richtigen Übungen auszuwählen.	B	3.65	1.03	-0.60	-0.14	0.54	
CCPT6	I know what to pay attention to in relation to my body in order to avoid excess load or insufficient load.	A	3.75	0.97	-0.66	0.25	0.54	
	Ich weiß, worauf ich bei meinem Körper achten muss, damit ich mich nicht über- oder unterfordere.	B	3.76	0.95	-0.60	0.08	0.55	
Mean Value for Scale CCPT		A	3.51	0.73	-0.29	-0.01		
		B	3.47	0.71	-0.27	-0.06		

PAAR1	I am able to regulate my mood through physical activity.	A	3.56	1.13	-0.56	-0.35	0.60	
	Ich bin in der Lage durch körperliche Aktivität meine Stimmung zu regulieren.	B	3.71	0.99	-0.59	0.05	0.66	
PAAR2	If I am feeling down, I can distract myself well through physical activity.	A	3.45	1.31	-0.40	-0.99	0.72	
	Wenn es mir schlecht geht, kann ich mich durch körperliche Aktivität gut ablenken.	B	3.38	1.28	-0.29	-1.02	0.76	
PAAR3	I am well able to improve my depressed mood by exercising.	A	3.49	1.21	-0.39	-0.76	0.75	0.85/ 0.88
	Mir gelingt es gut, meine gedrückte Stimmung durch Bewegung zu verbessern.	B	3.46	1.15	-0.42	-0.57	0.82	
PAAR4	I am well able to work off pent-up stress and inner tension through exercise.	A	3.61	1.20	-0.54	-0.59	0.67	
	Ich kann aufgestauten Stress und innere Anspannung durch Bewegung gut wieder abbauen.	B	3.57	1.15	-0.49	-0.57	0.75	
Mean Value for Scale PAAR		A	3.53	1.01	-0.44	-0.51		
		B	3.53	0.98	-0.42	-0.44		

Note. CCPT = Control competence for physical training; PAAR = Physical activity-specific affect regulation; M = Mean; SD = Standard Deviation; S = Skewness; K = Kurtosis; r_{it} = Corrected item-test-correlation; α = Cronbachs alpha.
