# Supplementary material for: Physical Activity-Related Health Competence, Physical Activity, and Physical Fitness: Analysis of Control Competence for the Self-Directed Exercise of Adolescents
Source: Int J Environ Res Public Health. 2019 Dec 19;17(1):39. doi: 10.3390/ijerph17010039 (PMC6981936; doi:10.3390/ijerph17010039)
Supplement: Supplementary file 1 [file ijerph-17-00039-s001.zip › Supplementary Material_PAHCO/Supplementary Material_Table S2.pdf]

Supplementary Material

Table S2. Mean values, standard deviation, and bivariate correlations of the relevant variables.

		N	*M*	*SD*	1	2	3	4	5	6	7	8
1	Cognitive attitudes towards the health effects of PA	832	4.65	0.44	-							
2	Affective attitudes towards the health effects of PA	832	3.84	0.75	0.48**	-						
3	Interest in training, physical fitness, and health	830	3.36	0.86	0.37**	0.54**	-					
4	Health-related fitness knowledge	834	0.00	1.26	0.14**	0.08*	0.19**	-				
5	Control competence for physical training	831	3.47	0.71	0.32**	0.44**	0.48**	0.10**	-			
6	Sport activity	811	4.92	4.06	0.24**	0.40**	0.29**	0.05	0.40**	-		
7	Cardiorespiratory fitness	782	0.39	0.83	0.21**	0.34**	0.26**	0.23**	0.27**	0.34**	-	
8	Muscular fitness	786	-0.56	0.85	0.19**	0.27**	0.23**	0.23**	0.26**	0.34**	0.51**	-

Note. N = sample size; *M* = Mean; *SD* = Standard Deviation.

* $p < 0.05$, ** $p < 0.001$.
